# Brief report: community-acquired Friedlander’s pneumonia and pulmonary metastatic *Klebsiella pneumoniae* infection caused by hypervirulent ST23 in the Netherlands

**DOI:** 10.1007/s10096-022-04470-z

**Published:** 2022-07-05

**Authors:** Monika Fliss, Charlotte H. S. B. van den Berg, Ed Kuijper, Daan W. Notermans, Antoni P. A. Hendrickx, Mirthe H. Schoots, Erik Bathoorn

**Affiliations:** 1 Department of Medical Microbiology, University of Groningen, University Medical Center Groningen, Groningen, Netherlands; 2 Department of Critical Care, University of Groningen, University Medical Center Groningen, Groningen, Netherlands; 3 Center for Infectious Disease Control, National Institute for Public Health and the Environment (RIVM), Bilthoven, Netherlands; 4 Department of Pathology and Medical Biology, University of Groningen, University Medical Center Groningen, Groningen, Netherlands

**Keywords:** Hypervirulent, Pneumonia, Klebsiella pneumonia, Friedlander, Carbapenemase

## Abstract

Infections with hypervirulent *Klebsiella pneumoniae* (hvKp) commonly presents with primary liver infection, bacteremia, and metastatic abscesses. Here, we present 2 cases of severe community-acquired pulmonary infections by hvKp in patients in the Netherlands without recent travel history. Both bacterial isolates are closely related to an archetype ST23 hvKp reference isolate. Based on these findings, surveillance programs on hvKp may consider to include isolates from community-acquired pneumonia by *K. pneumoniae*.

**Supplementary Information:**

The online version contains supplementary material available at 10.1007/s10096-022-04470-z.

## Introduction

Pulmonary infections by *K. pneumoniae* have first been described by the German pathologist/microbiologist Carl Friedlander in 1882. In 1889, the capsulated Bacillus of Friedlander, currently known as *K. pneumoniae*, was found as the causative agent. The disease, further named as Friedlander’s pneumonia, was characterized as a fulminant, lobular, and abscess-forming pneumonia caused by Friedlander’s bacillus [1]. The infection is community acquired in often debilitated patients with alcohol abuse, underlying pulmonary disease or immunocompromised status. The pneumonia most frequently involves the right upper lobe and has a fulminant course with a mortality rate of approximately 80% in the pre-antibiotic era. It has been hypothesized that this mucoid capsulated *K. pneumoniae* with a distinct severe pulmonary disease presentation can be considered a first recognition of a hypervirulent variant, but the associated genotypic characteristics and virulence genes have thus far not been described [2].

Hypervirulent *K. pneumoniae* (hvKp) also causes severe community-acquired disease. This disease entity was first described by Liu et al. in 1986 in Taiwan as primary liver abscess with metastatic spread to other organs such as the eye and brain by hvKp [3]. HvKp is associated with increased mucoviscosity of its colonies, although this is not fully pathognomic, and virulence genes involved in capsular synthesis regulation and iron acquisition. It is difficult to define hvKp at the molecular level. Based on experimental studies, the virulence of an isolate can be scored by detection of a defined set of virulence genes, as used in the Kleborate score [4]. Most of these genes are located on a hypervirulence plasmid. The ST23 lineage of *K. pneumoniae* is the archetype of hvKp [5].

An increasing number of reports from Asia but also from Europe have reported that hvKp strains have acquired carbapenemase genes. As a response to the emergence of carbapenemase producing hvKp, ECDC has reported a risk assessment with a request for surveillance on hvKp [6].

Here, we present two cases of severe community-acquired pulmonary infections with hypervirulent *K. pneumoniae* (hvKp) in patients in the Netherlands without recent travel history. Both bacterial isolates are closely related to each other and to an archetype ST23 hvKp reference isolate [4]. HvKp commonly presents with primary liver infection and metastatic abscesses, and molecularly confirmed pulmonary hvKp infections have scarcely been reported. Based on these findings, surveillance programs on hvKp may consider to include isolates from community-acquired pneumonia by *K. pneumoniae*.

## Cases

### Case of Friedlander’s pneumonia by hvKp

A 61-year-old Caucasian female presented with symptoms of coughing and dyspnea in May 2020 and was admitted to the internal medicine ward. She had been diagnosed with non-small cell lung cancer 4 months ago, for which she was treated with radiotherapy, which was complicated by radiation pneumonitis, and with the monoclonal antibody durvalumab as immunotherapy. She did not have a history of previous hospitalizations. She had complaints of coughing, with sometimes purulent sputum, and increasing dyspnea since 2 weeks, but no fever. X-ray imaging showed consolidations in the right middle and lower lobes. She was diagnosed with acute bronchopneumonia.

She was treated with oral amoxicillin/clavulanate 500/125 mg *t.i.d*, but the patient’s respiratory condition deteriorated during the first 5 days, and she was transferred to intensive care. Blood and bronchoalveolar lavage cultures grew hypermucoid *K. pneumoniae* (isolate UMCGhvKp1) with wild-type susceptibility based on automated susceptibility testing (VITEK2, bioMerieux, Marcy l’Etoile, France). The string test was positive: The colonies could be stretched in a string > 5 mm, which is indicative of increased mucoviscosity (Supplemental Fig. 1). An extensive panel of respiratory viruses including coronavirus SARS-2, influenza, parainfluenza, human metapneumovirus, and respiratory syncytial virus tested negative by PCR.

**Fig. 1:**
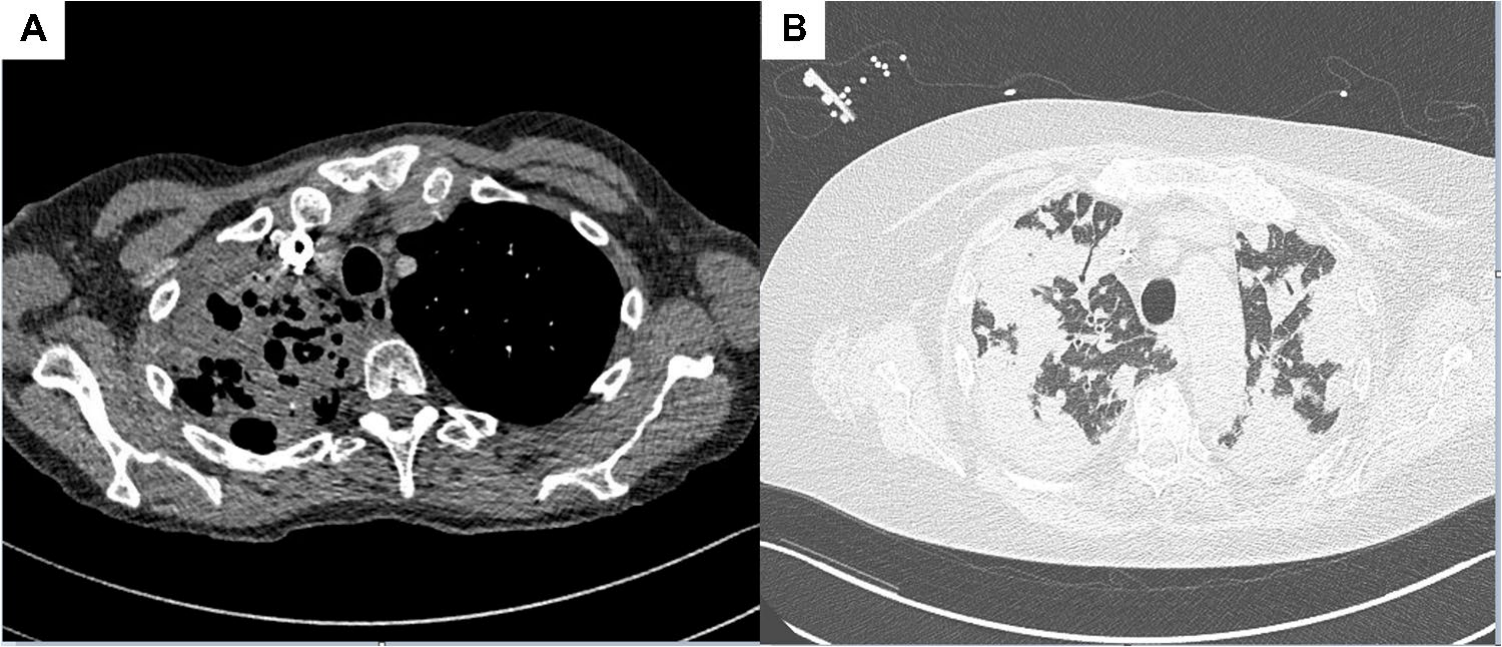
Computed tomography scans of **A** the first case showing consolidations in the right lung and a lung abscess in the right upper lung lobe and **B** the second case showing diffuse consolidations over all the lung fields

Computed tomography showed that the pneumonia had expanded to the right middle lobe, and a lung abscess had developed (Fig. 1A). The antibiotic regimen was switched to ceftriaxone 2 gr *q.d.* and ciprofloxacin 400 mg *t.i.d.*, and the patient showed good clinical response. On day 34 after admission, the patient was transferred to a pulmonary rehabilitation center.

### Case of a fatal pulmonary infection with metastatic abscesses by hvKp

A 74-year-old Caucasian female patient was admitted to the intensive care with respiratory insufficiency, low oxygen saturation, and a septic shock in November 2021. She had a history of steroid use for suspected polymyalgia rheumatica, total knee prosthesis, biliary pancreatitis, and recurrent urinary tract infections after previous bladder surgery. Her family reported that she had been in poor condition the past year and she seldomly went out of her house. In the past 2 weeks, she had complains of flu-like symptoms, fever, and muscle pain. On the evening of admission, she deteriorated and could not stand on her legs anymore. Pulmonary imaging showed diffuse spherical and confluent consolidations, but no abscesses (Fig. 1B). Blood, urine, and sputum cultures grew *K. pneumoniae* (isolate UMCGhvKp2) with wild-type susceptibility. The colonies were of hypermucoid morphology. The respiratory panel is described in case 1, and *Legionella pneumoniae*, *Mycoplasma pneumoniae*, *Chlamydia pneumoniae*, and *C. psittaci* tested negative by PCRs.

**Fig. 2:**
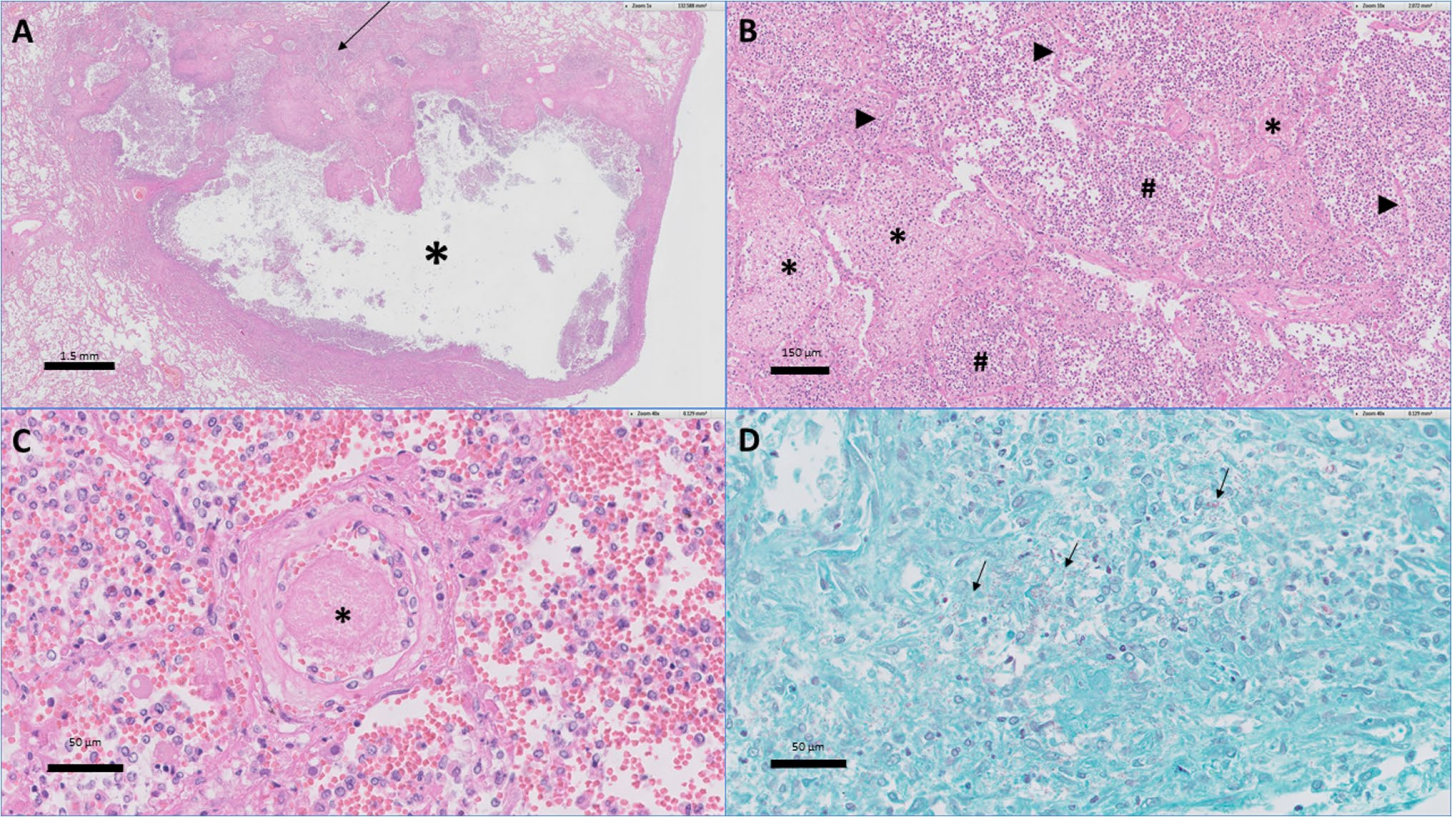
Lung histology of the second case in this study by Hematoxylin and Eosin staining (**A**–**C**) and Gram staining (**D**). **A** Lung overview with in the upper right and lower left relatively normal lung parenchyma. At the center, an abscess (*) is observed. Also, neutrophilic infiltrate and fibrin deposition (arrow) are shown, indicative of acute bronchopneumonia. **B** Close-up of the intra-alveolar neutrophilic infiltrates (#) with fibrin depositions (*) between the alveolar septa (arrowheads). The fibrin depositions and hyaline membrane development are indicative of diffuse alveolar damage. **C** In the center, a small vessel with a microthrombus is depicted (*). **D** Gram-negative rods in the lung parenchyma are depicted (arrows). Some of the rods are located intracellularly

Despite antibiotic therapy with cefotaxime 4 gr continuous infusion, tobramycin 7 mg/kg *o.d.* and doxycycline 200 mg, and optimal supportive care, the patient died of overwhelming septic shock with multi-organ failure, 15 h after admission. Autopsy showed abscesses in the right lung, right kidney, and surrounding perirenal fat. Histomorphology showed acute bronchopneumonia, infection of the lung parenchyma by Gram-negative rods, vasculitis, and focal microthrombi (Fig. 2). Also, in the right kidney, septic thrombi with secondary abscesses and pyelonephritis were seen. The right perirenal fat showed an abscess with neutrophilic infiltrate. Histomorphology of the bladder was not obtained, however, the pyelum of the right kidney showed neutrophilic infiltration of the urothelium indicative of a concurrent urinary tract infection.

In summary, we describe two patients with severe community-acquired pulmonary infections with wild type susceptible ST23 hvKp, the first presenting as a typical Friedlander’s pneumonia, and the second as septicemia with bronchopneumonia, urinary tract infection, and metastatic spread and development of pulmonary and (peri-)renal abscesses, respectively. Both cases first presented with clinical symptoms and imaging results in accordance with pneumonia, and the lung abscesses developed later during admission. In the first case, the initial oral treatment may have been insufficient to treat a pulmonary infection with *Klebsiella pneumonia* with an MIC of amoxicillin/clavulanate of 4 mg/L, which may have contributed to the clinical deterioration and positive blood cultures taken 5 days after start of antibiotic treatment. In the second case, we cannot be sure if the infection started with pneumonia or urinary tract infection.

Community acquired pneumonia with or without primary lung abscesses by hvKp has uncommonly been reported. A case study and literature review in 2020 reported only 10 cases in total [7]. Of these patients, 5 had bacteremia and 7 had septic shock. A high mortality rate of 50% of patients was reported. Diabetes was described as co-morbidity in 4 out of 10 cases. The reports in this review were from Japan, Taiwan, and South America. In Europe, a recent case of prostatic and lung abscesses without liver involvement has been reported from Spain. This patient was also diagnosed with diabetes type II as co-morbidity [8]. It is possible that the lung abscesses originated from a prostate abscess by metastatic spread, since the complaints started with anal pain.

In our study, both cases had important host risk factors for bacterial infections: The first patient had pulmonary damage by radiation pneumonitis and was treated with immunotherapy, and the second was immune compromised by prolonged corticosteroid use. Since both patients had not travelled recently, we conclude that hvKp was acquired in the Netherlands.

### Molecular characterization and comparison with hvKp isolates from Europe

Whole genome sequencing and hybrid de novo assembly were performed as described before, and the data is available from the European Nucleotide Archive bioproject PRJEB51459 [9]. The molecular characteristics of the two clinical *K. pneumoniae* isolates and a reference strain (SGH10) were analyzed and compared (Table 1). Molecular characterization by Pathogenwatch [10] revealed that both clinical isolates belong to MLST ST23, with K-locus 1 and O-locus V2. The presence of a KpVP-1 virulence plasmid and virulence genes encoding for yersiniabactin, colibactin, aerobactin, salmochelin, *rmpADC* type rmp 1, and *rmpA2* were detected in all isolates.

**Table 1:** Typing characteristics and virulence genes of the study isolates and the reference genome SGH10 as detected by the Kleborate web application [4]

	UMCGhvKp1	UMCGhvKp2	Sgh10
MLST and K/O-locus	ST23; K1/O2	ST23; K1/O2	ST23; K1/O2
*Yersiniabactin*	ybt1; YbST 45-1LV	ybt1; YbST47-4LV	ybt1; YbST 53
*Colibactin*	clb2; CbST 29	clb2; CbST29	clb2; CbST 29
*Aerobactin*	iuc1; AbST33	iuc1; AbST 1	iuc1; AbST 1
*Salmochelin*	iro1; SmST2	iro1; SmST 2	iro1; SmST 2
*RmpADC*	rmp1	rmp 1	rmp 1
*RmpA2*	rmpA2_6-55%	rmpA2_5-54%	rmpA2_6-47%
Kleborate score	5/5	5/5	5/5

**Fig. 3:**
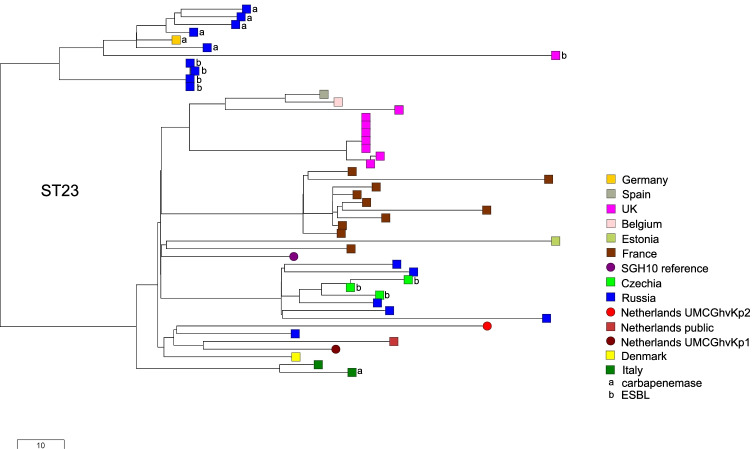
SNP-based neighbor-joining tree generated using the Pathogenwatch web application based on 1972 core genome genes of *K. pneumoniae* [8]. The study isolates are presented together with public available European *K. pneumoniae* isolates of MLST ST23. The corresponding isolate characteristics are shown in supplementary table1 in the same order as in the tree

A core genome SNP-based tree was created of the isolates in this study together with 45 publicly available *K. pneumoniae* isolates of ST23 from European countries in Pathogenwatch, with the presence of ESBL or carbapenemase genes marked (Fig. 3). The isolate characteristics are shown in Supplementary Table 1. All isolates in the tree are hvKp with a Kleborate score of 5, with exception of the isolate from Belgium and the carbapenemase-negative isolate from Italy. The isolates UMCGhvKp1 differed from UMCGhvKp2 by 97 SNPs in their core genome of 2,172,367 nucleotides. These isolates are located in the same clade with hvKp reference isolate SGH10, differing by 64 and 95 SNPs, respectively. Remarkably, the closest related isolate to UMCGhvKp1 differing by 58 SNPs is hvKp isolate SRR5713914, also from the Netherlands, isolated from human blood in 1997. Likewise, most of the other European isolates are clustering by country. It is possible that ST23 hvKp is present since many years and may have been unrecognized, since the archetype hvKp already was present in Europe in 1985 and has a common ancestor in the nineteenth century [5].

It is unsure from which source patients acquire hvKp infection in the community, but it may be a “One Health” problem. Among the public isolates in Fig. 1, the hvKp isolates of the cluster from France are also from horses, taken from a fetus and genital tract. *K. pneumoniae* with capsule type K1 is a causative pathogen of venereal disease in horses, detected in sperm, and in the genital tract in mares, causing metritis [11]. A recent study from Caribbean showed that the ST23 lineages is also found in vervets and horses [12]. The presence of hvKp in livestock is worrisome, since it can be transmitted via manure and soil water to food products. Indeed, this worst case scenario has already been reported by detection of carbapenemase-producing hvKp (cp-hvKp) in ready-to-eat-cucumber samples [13]. Unfortunately, no information is available of the two patients in this study about animal contacts and food consumption.

Cp-hvKp have been detected in many European countries including Ireland, France, Germany, Russia, Italy, and Spain [6, 14–16]. Almost all CPE producing hvKp isolates belong to the same clade within ST23, except for the VIM-producing hvKp from Italy (Fig. 3). The resistance to carbapenems and other classes of antibiotics is caused by acquisition of conjugative resistance plasmids. Several hospital outbreaks with cp-hvKp in Asia have been reported [6]. In addition, a nosocomial outbreak by OXA-48- and CTX-M-15-coproducing hvKp has been reported in Moscow [14]. The cp-hvKp hospital outbreaks are associated with high mortality [6]. This underlines that the hvKp lineage of ST23 forms a treat to both public health and patient care, requiring collaborative response from clinicians and public health institutions.

The recent observation of infections with hvKp ST23 carrying carbapenemase genes in EU/EEA countries further demonstrates the importance of recognition and surveillance programs to provide more insights in its prevalence and virulence. We alert clinicians and organizers of surveillance programs in public health that hvKp infections can present as community-acquired pulmonary infections with or without metastatic abscesses. Finally, we confirm that hvKp is endemic in the European community and a One Health approach is required in surveillance programs.

## Supplementary Information

Supplemental Figure 1 Positive string test of isolate UMCGhvKp1. The colonies could be stretched in a string >5 mm, which is associated with increased mucoviscosity. (PNG 2690 kb)

High resolution image (TIFF 1815 kb)

Supplemental Table 1 Characteristics of isolates from this study together with 45 public available *K pneumoniae* isolates of ST23 from European countries in Pathogenwatch accessed in February 2022.*: isolates from this study. (DOCX 20 kb)

## Data Availability

Nucleotide sequencing data of the study isolates is available from the European Nucleotide Archive bioproject PRJEB51459 at https://www.ebi.ac.uk/ena/browser/home. The data of the public isolates is available from the Pathogenwatch web application: https://pathogen.watch/, and the sample accession numbers are shown in the supplementary table. The data collection is reposited at https://pathogen.watch/genomes/all?collection=1riwk5is1rvt-europe2_hvkp&organismId=573.
