# Supplementary figures and images for: Brief report: community-acquired Friedlander’s pneumonia and pulmonary metastatic Klebsiella pneumoniae infection caused by hypervirulent ST23 in the Netherlands
Source: Eur J Clin Microbiol Infect Dis. 2022 Jul 5;41(8):1133–8. doi: 10.1007/s10096-022-04470-z (PMC9255504; doi:10.1007/s10096-022-04470-z)

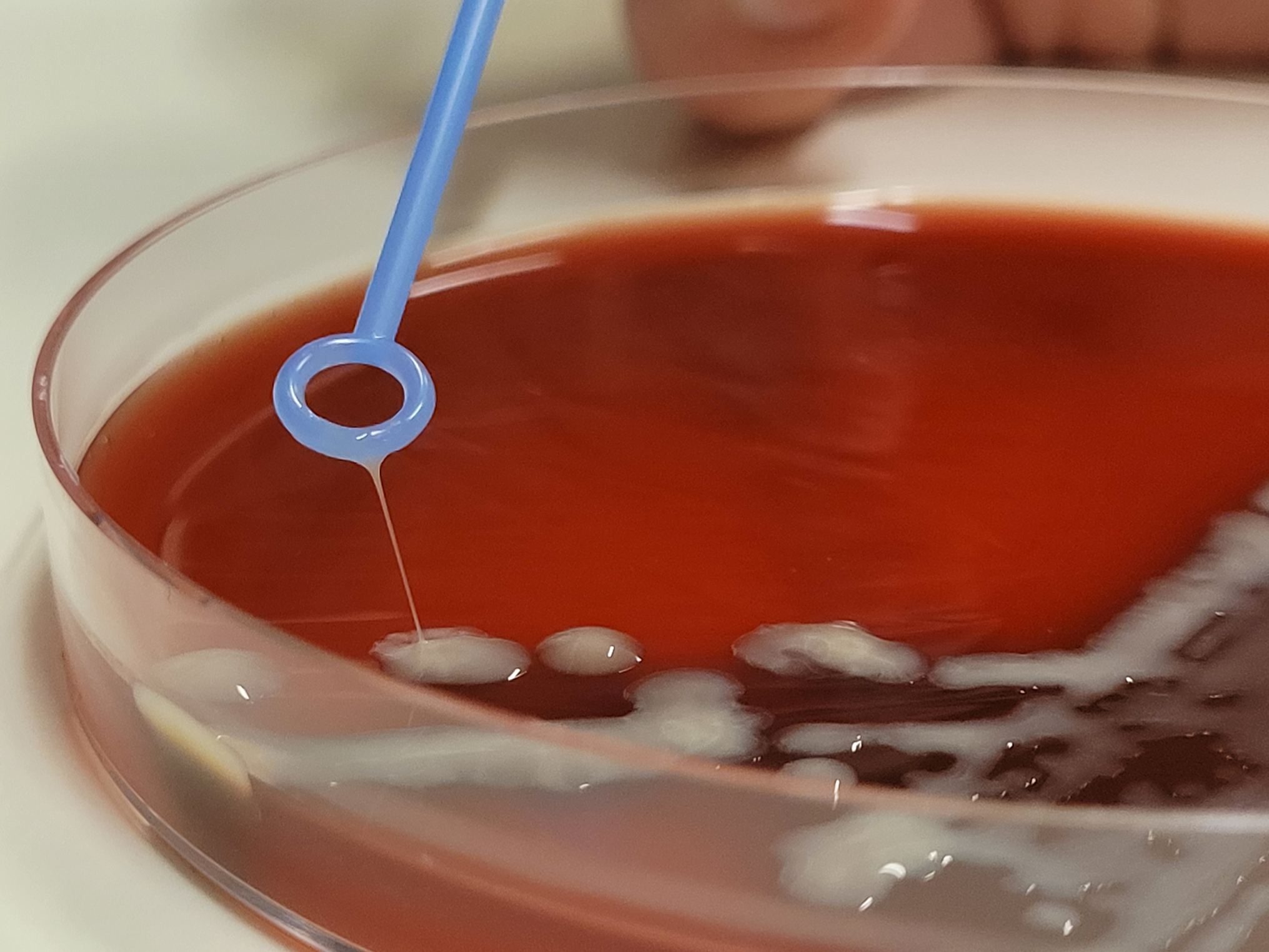

Supplement: Supplementary file 1 — Positive string test of isolate UMCGhvKp1. The colonies could be stretched in a string >5 mm, which is associated with increased mucoviscosity. (PNG 2690 kb) [file 10096_2022_4470_Fig4_ESM.png]

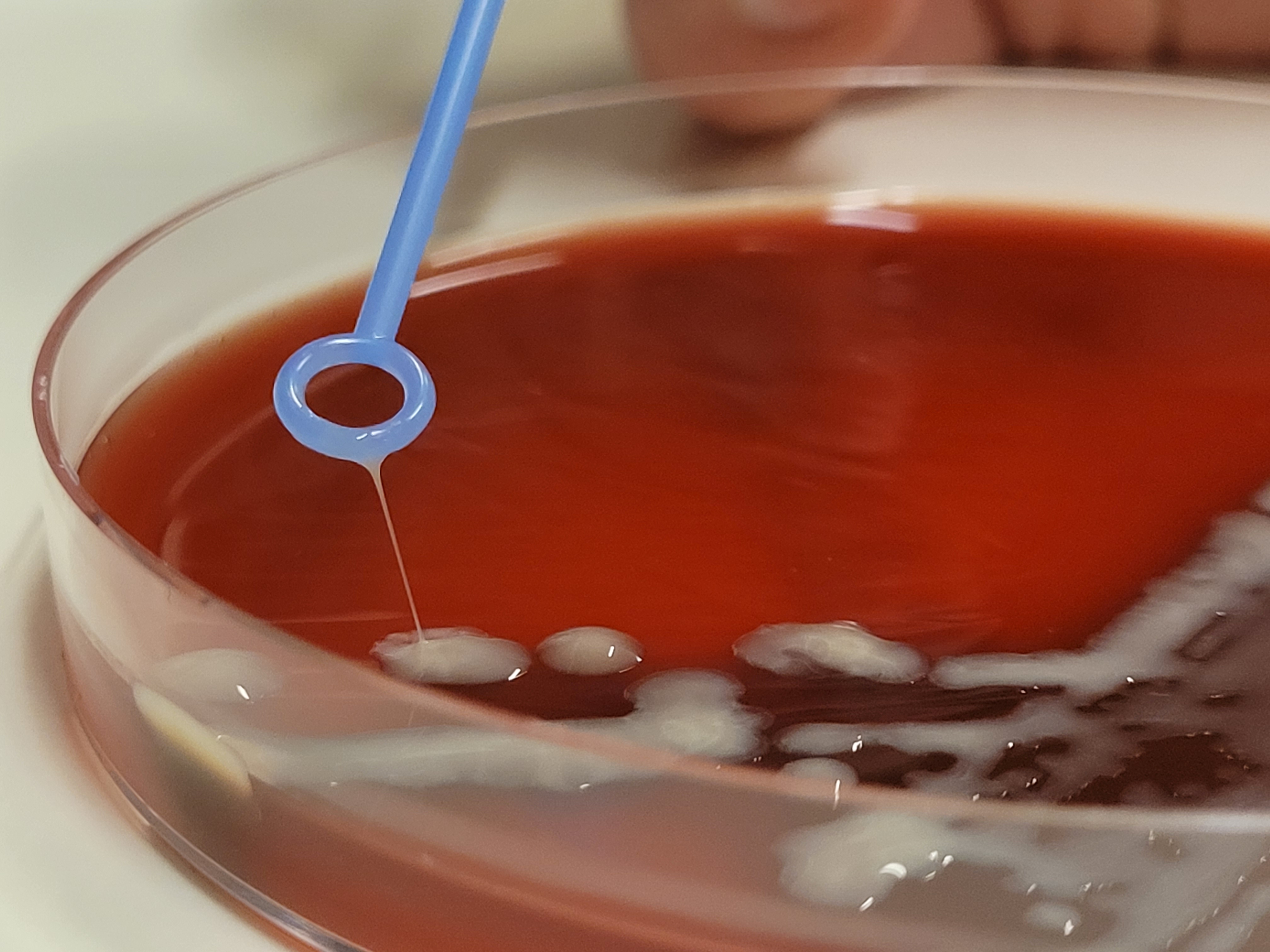

Supplement: Supplementary file 2 — High resolution image (TIFF 1815 kb) [file 10096_2022_4470_MOESM1_ESM.tiff]
