# Supplementary material for: Brief report: community-acquired Friedlander’s pneumonia and pulmonary metastatic Klebsiella pneumoniae infection caused by hypervirulent ST23 in the Netherlands
Source: Eur J Clin Microbiol Infect Dis. 2022 Jul 5;41(8):1133–8. doi: 10.1007/s10096-022-04470-z (PMC9255504; doi:10.1007/s10096-022-04470-z)
Supplement: Supplementary file 3 — Characteristics of isolates from this study together with 45 public available K pneumoniae isolates of ST23 from European countries in Pathogenwatch accessed in February 2022.*: isolates from this study. (DOCX 20 kb) [file 10096_2022_4470_MOESM2_ESM.docx]

Supplementary table 1: Isolate characteristics

| **Sample** | **Year** | **Sample accession** | **Country** | **Host** | **Isolation source** | **Carbapenemase or ESBL** |
| --- | --- | --- | --- | --- | --- | --- |
| SRR9208899 | 2014 | SAMN11878536 | Russia | Human | Wound | OXA-48; CTX-M-15 |
| SRR9208900 | 2012 | SAMN11878535 | Russia | Human | Sputum | OXA-48; CTX-M-15 |
| SRR9208898 | 2014 | SAMN11878533 | Russia | Human | Urine | OXA-48; CTX-M-15 |
| SRR9208903 | 2014 | SAMN11878532 | Russia | Human | Urine | OXA-48; CTX-M-15 |
| ERR1761497 | 2012 | SAMEA29232418 | Germany | Human | Tracheal secretion | OXA-48; CTX-M-15 |
| SRR9208902 | 2013 | SAMN11878529 | Russia | Human | Aspirate | OXA-48; CTX-M-15 |
| SRR9208896 | 2013 | SAMN11878537 | Russia | Human | Unknown | CTX-M-15 |
| SRR12102925 | 2017 | SAMN12349650 | Russia | Human | Faeces | CTX-M-15 |
| SRR12102926 | 2017 | SAMN12349649 | Russia | Human | Faeces | CTX-M-15 |
| SRR12102921 | 2017 | SAMN12349654 | Russia | Human | Faeces | CTX-M-15 |
| SRR12102922 | 2017 | SAMN12349653 | Russia | Human | Faeces | CTX-M-15 |
| SRR5713912 | 1997 | SAMN07256686 | Spain | Human | Blood |  |
| SRR5713916 | 1997 | SAMN07256672 | Belgium | Human | Blood |  |
| ERR257665 | 2008 | SAMEA1712950 | United Kingdom | Human | Unknown |  |
| ERR1796031 | 2015 | SAMEA4362688 | United Kingdom | Environmental | Environment |  |
| ERR1796027 | 2015 | SAMEA4362684 | United Kingdom | Environmental | Environment |  |
| ERR1796032 | 2015 | SAMEA4362689 | United Kingdom | Environmental | Environment |  |
| ERR1796033 | 2015 | SAMEA4362690 | United Kingdom | Environmental | Environment |  |
| ERR1796029 | 2015 | SAMEA4362686 | United Kingdom | Environmental | Environment |  |
| ERR1796028 | 2015 | SAMEA4362685 | United Kingdom | Environmental | Environment |  |
| ERR1796026 | 2015 | SAMEA4362683 | United Kingdom | Environmental | Environment |  |
| SRR5713920 | 1985 | SAMN07256676 | France | Equus caballus | Genital tract |  |
| SRR5713913 | 1980 | SAMN07256675 | France | Equus caballus | Genital tract |  |
| SRR5713908 | 1987 | SAMN07256683 | France | Equus caballus | Genital tract |  |
| SRR5713910 | 1987 | SAMN07256685 | France | Equus caballus | Stallion sperm |  |
| SRR5713922 | 1985 | SAMN07256680 | France | Equus caballus | Foetus |  |
| SRR5713918 | 1985 | SAMN07256678 | France | Equus caballus | Genital tract |  |
| SRR5713909 | 1987 | SAMN07256682 | France | Equus caballus | Cervix |  |
| SRR5713917 | 1985 | SAMN07256679 | France | Equus caballus | Genital tract |  |
| SRR5713915 | 1985 | SAMN07256673 | France | Equus caballus | Unknown |  |
| ERR1205002 | 2013 | SAMEA3499911 | Estonia | Human | Lower respiratory tract secrection |  |
| SRR8738428 | 2008 | SAMN11125719 | France | Human | Unknown |  |
| SGH10 | 2014 | SAMN06112188 | Singapore | Human | Liver abscess |  |
| SRR9208897 | 2013 | SAMN11878534 | Russia | Human | Aspirate |  |
| SRR9208904 | 2015 | SAMN11878531 | Russia | Human | Unknown |  |
| SRR8452425 | 2014 | SAMN10763118 | Czech Republic | Human | Rectum | CTX-M-15 |
| SRR8452419 | 2014 | SAMN10763117 | Czech Republic | Human | Blood culture | CTX-M-15 |
| SRR5137059 | 2014 | SAMN06190126 | Czech Republic | Human | Wound swab | CTX-M-15 |
| SRR9208901 | 2014 | SAMN11878530 | Russia | Human | Unknown |  |
| SRR12102923 | 2017 | SAMN12349652 | Russia | Human | Faeces |  |
| SRR5432530 | 2003 | SAMN06606879 | Russia | Human | Faeces |  |
| UMCGhvKp1* | 2020 | SAMEA14252881 | Netherlands | Human | Blood |  |
| SRR5713914 | 1997 | SAMN07256674 | Netherlands | Human | Blood |  |
| SRR9208895 | 2016 | SAMN11878538 | Russia | Human | Unknown |  |
| UMCGhvKp2* | 2021 | SAMEA14252882 | Netherlands | Human | Bronchoalveolar lavage |  |
| SRR4036806 | Unknown | SAMN05581771 | Denmark | Unknown | Unknown |  |
| ERR1217325 | 2014 | SAMEA3515178 | Italy | Human | Other (drainage) |  |
| ERR3164635 | 2016 | SAMEA104414832 | Italy | Human | Unknown | VIM-1 |

Characteristics of isolates from this study together with 45 public available *K pneumoniae* isolates of ST23 from European countries in Pathogenwatch accessed in February 2022.*: isolates form this study.
